# Piezo1 as a Key Mechanosensitive Ion Channel Linking Mechanical Overload to Mitochondrial Dysfunction, Mitophagy, and Immunometabolic Dysregulation in Osteoarthritis

**DOI:** 10.3390/cells15171511

**Published:** 2026-08-22

**Authors:** Hechmi Toumi, Ahmad Almhdie-Imjabbar, Eric Lespessailles

**Affiliations:** 1 Translational Medicine Research Platform, PRIMMO, University Hospital Center of Orleans, 14 Avenue de l’Hôpital, 45100 Orleans, France; eric.lespessailles@chu-orleans.fr; 2 Department of Rheumatology, University Hospital Center of Orleans, 14 Avenue de l’Hôpital, 45100 Orleans, France; 3 Faculty of Sciences and Technology, University of Orleans, 1 Rue de Chartres, 45100 Orleans, France; 4 Laboratory of Metabolic Adaptations to Exercise in Physiological and Pathological Conditions (AME2P), Clermont Auvergne University (UCA), 3 Rue de la Chebarde, 63178 Aubière, France

**Keywords:** osteoarthritis, Piezo1, mechanotransduction, mitochondria, mitochondrial dysfunction, mitophagy, immunometabolism, calcium signaling, precision medicine

## Abstract

**Highlights:**

**What are the main findings?**

- Piezo1 is a central mechanosensor linking pathological mechanical loading to mitochondrial dysfunction, impaired mitophagy, and immunometabolic dysregulation in osteoarthritis.
- Persistent Piezo1 activation may initiate a self-amplifying cascade involving calcium overload, mitochondrial injury, cGAS–STING and NLRP3 activation, thereby contributing to cartilage degeneration.

**What are the implications of the main findings?**

- Targeting the Piezo1–mitochondria–immune axis represents a promising strategy for developing disease-modifying therapies for osteoarthritis.
- Integrating mechanobiology, mitochondrial medicine, and precision-targeted delivery systems may accelerate the translation of next-generation therapeutic approaches into clinical practice.

**Abstract:**

Osteoarthritis (OA) is increasingly recognized as a mechanically driven whole-joint disease in which abnormal mechanotransduction initiates a cascade of mitochondrial dysfunction, chronic inflammation, and progressive cartilage degeneration. Among the mechanosensitive molecules identified to date, Piezo1 has emerged as a key mechanosensitive regulator linking pathological mechanical loading to intracellular calcium signaling and downstream cellular responses. Growing evidence indicates that persistent Piezo1 activation promotes mitochondrial calcium overload, excessive reactive oxygen species production, ATP depletion, mitochondrial membrane depolarization, and impaired mitophagy, ultimately amplifying chondrocyte dysfunction and extracellular matrix degradation. In parallel, mitochondrial damage triggers immunometabolic reprogramming through activation of the cGAS–STING pathway and the NLRP3 inflammasome. It also promotes pro-inflammatory cytokines, including interleukin-1β, tumor necrosis factor-α, and interleukin-6. Together, these responses may contribute to a self-perpetuating cycle of inflammation and tissue destruction. This review provides a comprehensive synthesis of recent advances regarding the role of Piezo1 in OA, focusing on the mechanistic links between mechanotransduction, mitochondrial dysfunction, mitophagy, and immunometabolic dysregulation. We further discuss the contribution of mitochondrial quality-control pathways, including PINK1/Parkin-, BNIP3-, and FUNDC1-mediated mitophagy, as well as alterations in mitochondrial dynamics involving DRP1, MFN1, MFN2, and OPA1. Emerging experimental models are discussed as valuable tools for accelerating therapeutic discovery. Finally, we critically evaluate current therapeutic strategies targeting the Piezo1–mitochondria axis, including mechanosensitive channel modulation, mitochondrial protection, mitophagy enhancement, gene therapy, biomaterial-assisted delivery, and nanomedicine. Collectively, current evidence supports the Piezo1–mitochondria–immune axis as an important mechanistic framework contributing to OA pathogenesis and as a potential therapeutic target. Integrating mechanobiology, mitochondrial medicine, and precision-engineered experimental models may facilitate the development of next-generation disease-modifying therapies capable of slowing or preventing osteoarthritis progression.

## 1. Introduction

Osteoarthritis (OA) is the most prevalent chronic joint disease and a leading cause of pain, disability, and reduced quality of life worldwide. Traditionally regarded as a passive consequence of aging and mechanical wear, OA is now recognized as a complex whole-joint disorder involving dynamic interactions among articular cartilage, subchondral bone, synovium, menisci, ligaments, and periarticular muscles. Accumulating evidence indicates that biomechanical abnormalities, low-grade inflammation, oxidative stress, metabolic dysfunction, mitochondrial dysfunction, and cellular senescence collectively drive disease initiation and progression rather than acting as independent pathogenic mechanisms [1,2]. Despite substantial advances in understanding OA pathophysiology, current treatments remain largely symptomatic, and no disease-modifying osteoarthritis drug (DMOAD) has demonstrated consistent long-term clinical efficacy. Consequently, identifying molecular mechanisms that couple abnormal mechanical stimuli with inflammatory and metabolic responses has become a major research priority.

Mechanical overload is increasingly recognized as one of the earliest drivers of OA initiation and progression. Under physiological conditions, mechanical forces are essential for maintaining cartilage integrity, extracellular matrix (ECM) turnover, and chondrocyte homeostasis. However, excessive or repetitive loading can result from obesity, joint malalignment, trauma, ligament instability, or age-related matrix stiffening. Such loading disrupts cartilage mechanoadaptation and activates catabolic pathways, leading to ECM degradation, oxidative stress, inflammatory responses, and progressive cartilage destruction [3,4,5]. These findings have shifted the current view of OA from a passive degenerative disorder to a mechanically driven disease in which dysregulated mechanotransduction represents a central pathogenic mechanism.

Mechanotransduction, the process by which cells convert mechanical stimuli into biochemical signals, is fundamental to cartilage physiology and adaptation. Chondrocytes sense mechanical cues through integrins, the cytoskeleton, primary cilia, transient receptor potential (TRP) channels, and mechanically activated ion channels. Among these mechanosensors, Piezo1 has emerged as a key regulator of mechanically induced calcium signaling because it directly converts membrane deformation into rapid Ca^2+^ influx. Since its discovery by Coste et al. [6], Piezo1 has been implicated in vascular physiology, erythrocyte volume regulation, skeletal mechanobiology, and bone remodeling. More recently, accumulating evidence has identified Piezo1 as a critical mediator of OA, where its persistent activation promotes calcium overload, extracellular matrix degradation, inflammatory signaling, and chondrocyte death [5,7,8].

Beyond its role in calcium signaling, Piezo1 has emerged as a critical regulator of mitochondrial homeostasis. Persistent Ca^2+^ influx induced by mechanical overload promotes mitochondrial calcium accumulation, excessive reactive oxygen species (ROS) production, adenosine triphosphate (ATP) depletion, and mitochondrial membrane depolarization, ultimately compromising cellular bioenergetics. As central regulators of energy metabolism, redox balance, and innate immunity, dysfunctional mitochondria have become recognized as key contributors to OA progression. Increasing evidence indicates that mitochondrial dysfunction represents a major downstream consequence of sustained Piezo1 activation, linking abnormal mechanotransduction to progressive cartilage degeneration [8,9,10,11].

Maintenance of mitochondrial integrity relies on efficient quality-control mechanisms, particularly mitophagy, which selectively eliminates dysfunctional mitochondria to preserve cellular homeostasis. Under persistent mechanical overload, sustained Piezo1 activation appears to overwhelm these protective pathways, resulting in defective mitophagy, accumulation of damaged mitochondria, and amplification of oxidative stress. Mitochondrial injury further promotes immunometabolic dysregulation through the release of mitochondrial ROS and mitochondrial DNA (mtDNA), leading to activation of the cyclic GMP–AMP synthase (cGAS)–stimulator of interferon genes (STING) pathway, the NOD-like receptor family pyrin domain containing 3 (NLRP3) inflammasome, and nuclear factor kappa B (NF-κB) signaling. Together, these events may contribute to a self-perpetuating cycle linking mechanical overload to chronic inflammation and progressive cartilage degeneration [8,12,13,14].

Collectively, these findings support a central mechanobiological role for Piezo1 in connecting mechanical overload, mitochondrial dysfunction, mitophagy impairment, and immunometabolic remodeling during OA progression. Nevertheless, several key questions remain unresolved. These include the precise molecular mechanisms linking Piezo1 to mitochondrial quality control, the cell-specific functions of Piezo1 within the osteoarthritic joint, and the therapeutic potential of targeting the Piezo1–mitochondria axis.

In this review, we summarize recent advances in Piezo1 biology and discuss how abnormal mechanotransduction may contribute to mitochondrial dysfunction, defective mitophagy, and immunometabolic dysregulation in osteoarthritis. We also critically examine emerging therapeutic strategies targeting the Piezo1–mitochondria axis and highlight future perspectives for the development of disease-modifying interventions. Importantly, current mechanistic evidence is strongest in articular chondrocytes. However, Piezo1 is also expressed across multiple joint-associated cell populations, including synovial fibroblasts, macrophages, osteoblasts, and osteocytes. Piezo1-dependent mechanotransduction may therefore contribute to OA as a whole-joint process rather than solely as a cartilage-centered mechanism.

While previous reviews have primarily addressed Piezo1-mediated mechanotransduction and its contribution to cartilage degeneration, the present review extends this framework by integrating Piezo1 signaling with mitochondrial dysfunction, mitochondrial quality control, mitophagy, and immunometabolic regulation. We propose the Piezo1–mitochondria–immune axis as an integrative conceptual framework linking abnormal mechanical loading to mitochondrial stress, defective mitophagy, innate immune activation, and progressive joint degeneration. Rather than representing a newly established linear pathway, this framework synthesizes currently fragmented mechanobiological, mitochondrial, and immunometabolic evidence and highlights potential intervention points for disease-modifying strategies in OA.

**Figure 1:**
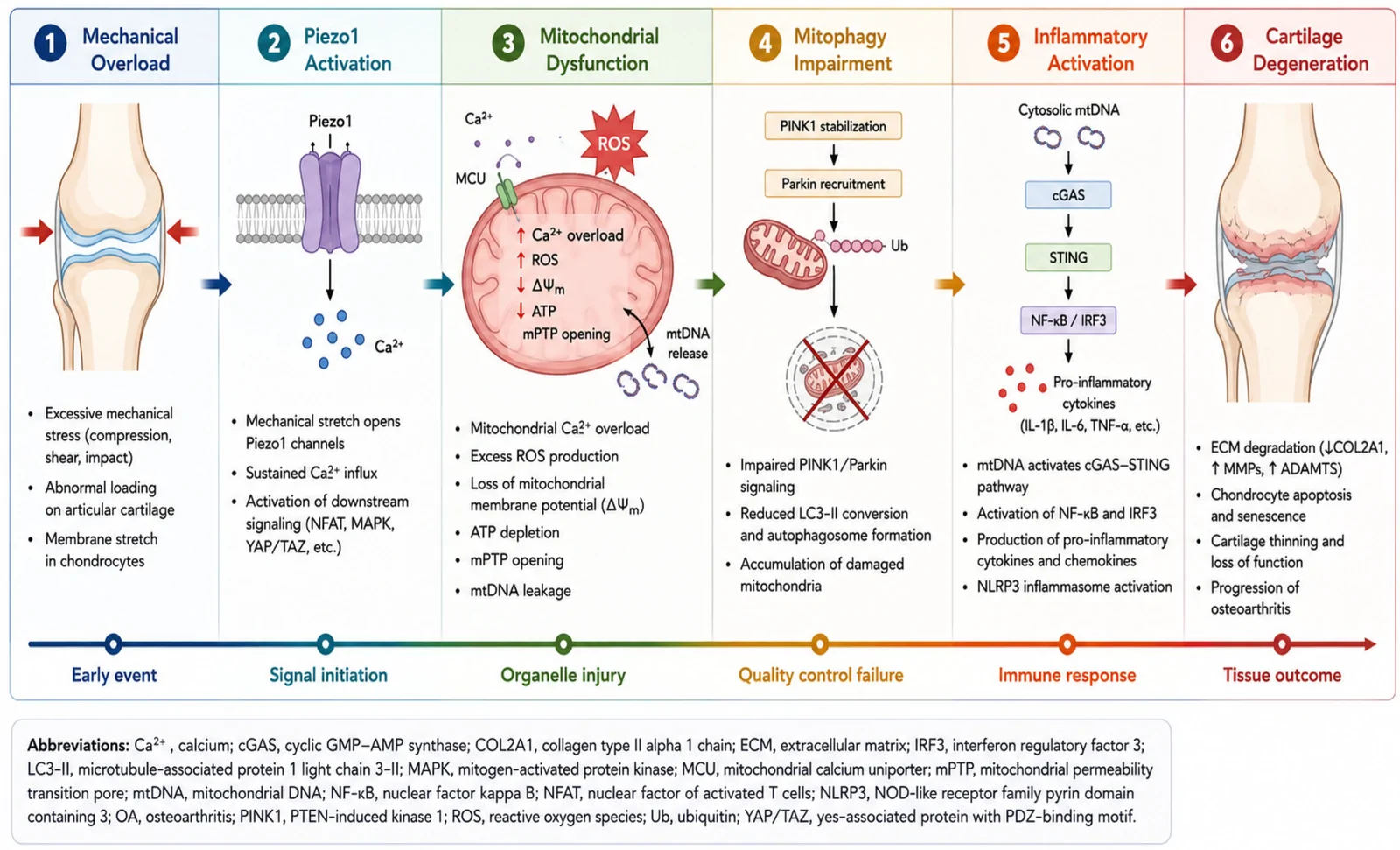
Proposed mechanistic timeline of the Piezo1–mitochondria–mitophagy–inflammation axis in osteoarthritis. Chronic mechanical overload promotes Piezo1 activation and sustained Ca^2+^ influx, which may contribute to mitochondrial Ca^2+^ overload, oxidative stress, loss of mitochondrial membrane potential, ATP depletion, and mitochondrial DNA release. Mitochondrial dysfunction is associated with impaired mitophagy and accumulation of damaged mitochondria, thereby promoting innate immune and inflammatory signaling. These interconnected processes ultimately contribute to chondrocyte dysfunction, extracellular matrix degradation, and osteoarthritis progression. Arrows indicate the direction and sequential progression of the proposed mechanistic pathway, while the color coding distinguishes the successive stages of the cascade, from the early mechanical event and Piezo1-mediated signal initiation to mitochondrial injury, mitophagy impairment, inflammatory activation, and the final cartilage tissue outcome. The sequence shown represents an integrated mechanistic framework based predominantly on preclinical evidence and should not be interpreted as an established linear causal pathway in human osteoarthritis.

To provide an integrated overview of the mechanistic framework developed in this review, Figure 1 summarizes the proposed sequential progression from mechanical overload to Piezo1 activation, mitochondrial dysfunction, mitophagy impairment, inflammatory activation, and ultimately cartilage degeneration. This timeline provides a conceptual framework for the detailed molecular mechanisms discussed in the subsequent sections.

## 2. Methods

### 2.1. Literature Search Strategy

A structured literature search was conducted using the PubMed/MEDLINE database to identify studies investigating the role of Piezo1 in osteoarthritis, with particular emphasis on mechanotransduction, mitochondrial dysfunction, mitophagy, immunometabolic regulation, and cartilage biology. Publications indexed between January 2020 and May 2026 were considered.

The following search strategy was applied:

(“Osteoarthritis”[Mesh] OR osteoarthritis[Title/Abstract])

AND

(Piezo1[Title/Abstract] OR mechanotransduction[Title/Abstract] OR mechanobiology[Title/Abstract] OR “mechanosensitive ion channel*”[Title/Abstract])

AND

(“Chondrocytes”[Mesh] OR chondrocyte*[Title/Abstract] OR cartilage[Title/Abstract])

AND

(“1 January 2020”[Date—Publication]: “3000”[Date—Publication])

The search identified 221 records, which were screened according to their relevance to the objectives of this review. A final literature update was performed before resubmission to identify relevant studies published or indexed since the initial search, including newly available studies from 2026. Newly identified studies relevant to Piezo1-mediated mechanotransduction, mitochondrial dysfunction, mitophagy, immunometabolic signaling, and the distinction between Piezo1 and Piezo2 were evaluated and incorporated as complementary references where appropriate. Four additional complementary references were added during this update [15,16,17,18]. These references were not part of the formally selected study corpus and therefore did not modify the PRISMA study count of 47 original studies.

### 2.2. Eligibility Criteria

Studies were eligible if they investigated one or more of the following topics:

Piezo1 biology and mechanotransduction in osteoarthritis;

mechanical loading and cartilage mechanobiology;

mitochondrial dysfunction and calcium signaling downstream of Piezo1;

mitophagy and mitochondrial quality control;

immunometabolic regulation and inflammatory signaling;

therapeutic strategies targeting the Piezo1–mitochondria axis.

Review articles, editorials, conference abstracts, non-English publications, and studies unrelated to osteoarthritis or cartilage biology were excluded.

### 2.3. Study Selection

**Figure 2:**
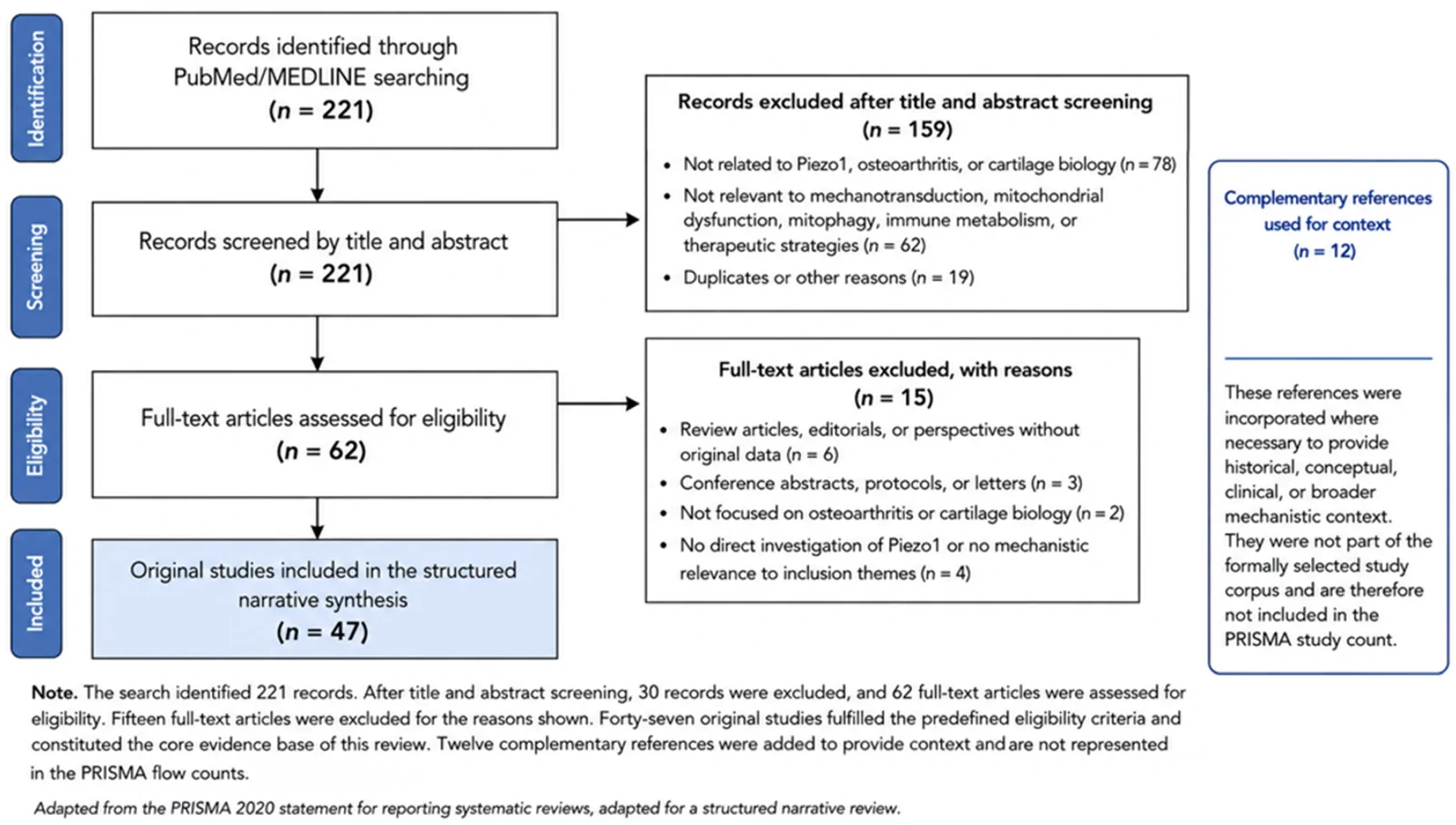
Literature search and study selection process for the structured narrative review. Forty-seven original studies fulfilled the predefined eligibility criteria and were included in the structured narrative synthesis. Additional references used for historical, conceptual, clinical, or broader mechanistic context were not part of the formal study selection process and are therefore not represented in the PRISMA flow diagram. Adapted from the PRISMA 2020 framework for a structured narrative review.

Titles and abstracts were screened to assess their relevance to the objectives of this review. Studies directly addressing the predefined topics were included in the qualitative synthesis, whereas articles outside the scope of the review were excluded. Following screening and eligibility assessment, 47 original studies fulfilled the predefined inclusion criteria and constituted the core evidence base of this review. Twelve complementary references were incorporated to provide historical, conceptual, clinical, or broader mechanistic context [1,2,6,9,11,12,19,20,21,22,23,24]. A subsequent literature update before resubmission added four further complementary references [15,16,17,18]. Thus, the reference list contains 63 references in total, whereas the formally selected PRISMA corpus remains 47 original studies. The complementary references were not included in the PRISMA study count. The literature search strategy and study selection process are summarized in Figure 2.

### 2.4. Data Extraction and Evidence Synthesis

For each included study, information was extracted regarding experimental models, Piezo1-related signaling pathways, mitochondrial alterations, mitophagy, immunometabolic responses, therapeutic interventions, and principal findings. The evidence was synthesized into five thematic sections: (i) Piezo1 biology and mechanotransduction, (ii) mitochondrial dysfunction, (iii) mitophagy, (iv) immunometabolic dysregulation, and (v) therapeutic opportunities targeting the Piezo1–mitochondria axis. Because this manuscript is a narrative review, no quantitative meta-analysis was performed.

The 47 studies included in this review (Table 1) were classified according to their primary scientific contribution within one of the five major thematic sections of the manuscript. Core studies represent the principal evidence supporting each section, whereas supporting studies provide complementary mechanistic, biological, or translational evidence. Studies that addressed broader aspects of osteoarthritis biology or Piezo1 signaling, but did not fit predominantly within one of these five themes, were grouped under Other Relevant Topics. Accordingly, not all included studies are discussed in dedicated sections of the review, although all contributed to the overall narrative synthesis.

To facilitate critical appraisal of the heterogeneous evidence base, representative studies were additionally summarized according to experimental model, species or biological source, intervention or exposure, major findings, and evidence category (Table 2). Evidence categories were defined descriptively according to study design. They included human genetic/mechanistic evidence, human tissue or in vitro evidence, human ex vivo evidence, in vivo preclinical evidence, in vitro mechanistic evidence, multi-model mechanistic/preclinical evidence, omics evidence, and biomaterial evidence. These categories were not intended to represent a formal clinical hierarchy of evidence.

## 3. Piezo1: A Key Mechanosensitive Ion Channel in Cartilage

### 3.1. Piezo1 Biology: Structure, Activation and Mechanical Gating

**Figure 3:**
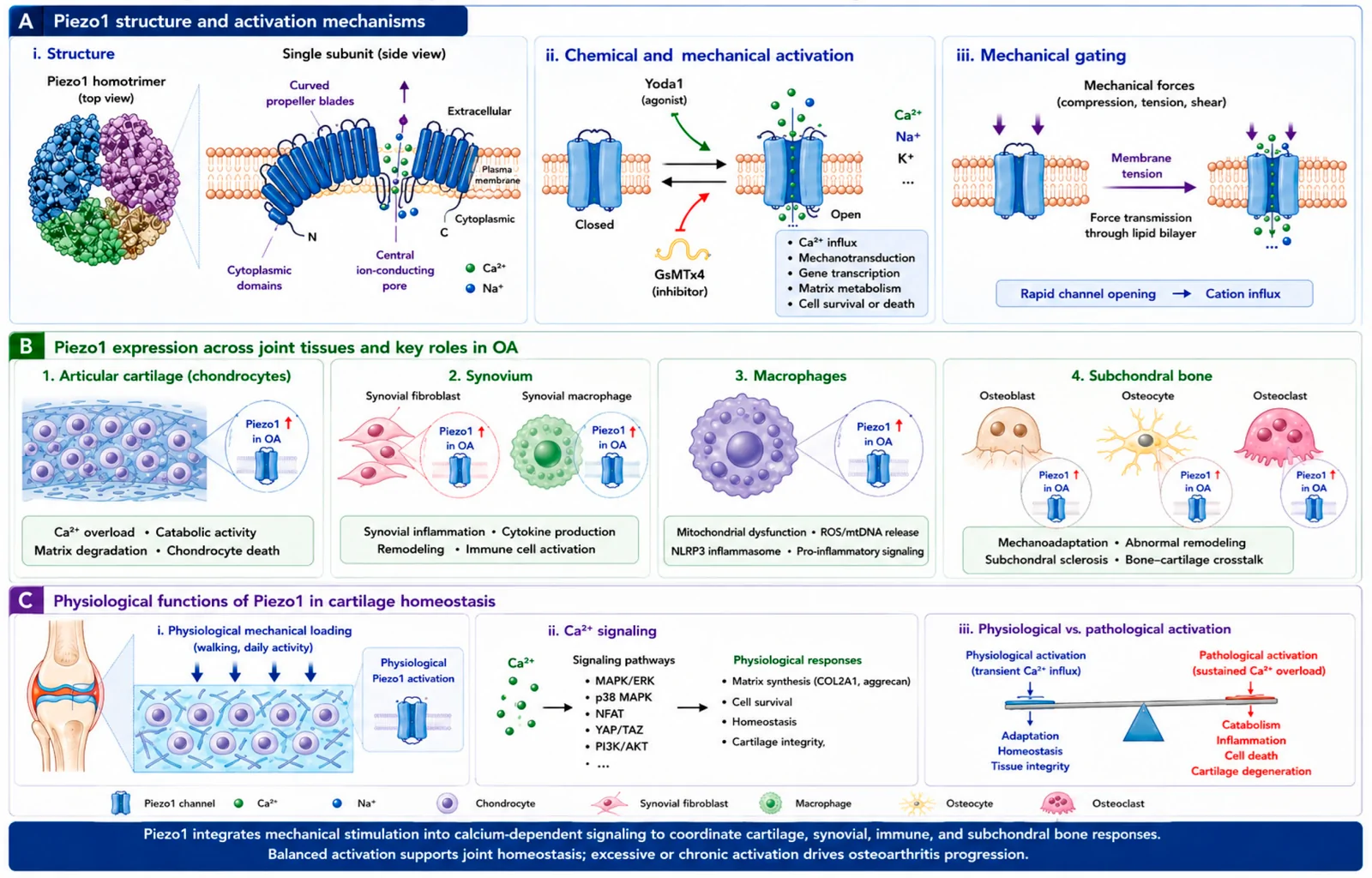
Piezo1 biology and mechanotransduction in cartilage homeostasis and osteoarthritis (OA). (**A**) Piezo1 structure and activation mechanisms. Piezo1 forms a homotrimeric mechanically activated cation channel that can be activated by mechanical forces or pharmacologically by Yoda1, whereas GsMTx4 acts as a mechanosensitive channel inhibitor. Channel opening promotes cation influx, particularly Ca^2+^, and initiates downstream mechanotransduction signaling. (**B**) Piezo1 expression and key roles across joint tissues in OA. Piezo1 is expressed in articular chondrocytes, synovial fibroblasts and macrophages, and subchondral bone cells, where excessive activation may contribute to Ca^2+^ overload, matrix degradation, inflammatory signaling, mitochondrial dysfunction, and abnormal bone remodeling. (**C**) Physiological functions of Piezo1 in cartilage homeostasis. Transient Piezo1 activation during physiological mechanical loading supports Ca^2+^-dependent signaling, matrix synthesis, cell survival, and tissue homeostasis, whereas sustained or excessive activation shifts mechanotransduction toward catabolism, inflammation, cell death, and cartilage degeneration. Abbreviations: ADAMTSs, a disintegrin and metalloproteinase with thrombospondin motifs; AKT, protein kinase B; Ca^2+^, calcium; COL2A1, collagen type II alpha 1 chain; ERK, extracellular signal-regulated kinase; GsMTx4, Grammostola spatulata mechanotoxin 4; K^+^, potassium; MAPK, mitogen-activated protein kinase; MMPs, matrix metalloproteinases; mtDNA, mitochondrial DNA; Na^+^, sodium; NFAT, nuclear factor of activated T cells; NLRP3, NOD-like receptor family pyrin domain containing 3; OA, osteoarthritis; PI3K, phosphoinositide 3-kinase; ROS, reactive oxygen species; YAP/TAZ, yes-associated protein/transcriptional coactivator with PDZ-binding motif; Yoda1, Piezo1 agonist.

Mechanotransduction is essential for maintaining articular cartilage homeostasis by enabling chondrocytes to sense and respond to changes in their mechanical environment. Among the mechanosensitive proteins identified to date, Piezo1 has emerged as a central regulator of this process. Since its identification in 2010 [6], Piezo1 has been recognized as a mechanically activated, non-selective cation channel that directly converts membrane deformation into rapid Ca^2+^ influx. Structurally, Piezo1 forms a homotrimeric propeller-shaped complex embedded within the plasma membrane. Mechanical tension induces conformational changes that open the central ion-conducting pore, initiating calcium-dependent signaling pathways involved in cellular adaptation, extracellular matrix homeostasis, and mechanobiological responses. Unlike ligand- or voltage-gated ion channels, Piezo1 is activated directly by membrane tension, allowing cells to rapidly detect compressive, tensile, and shear forces. Under physiological conditions, transient Piezo1 activation supports cartilage homeostasis, whereas persistent mechanical overload results in sustained calcium influx and initiates the downstream pathological mechanisms discussed in the following sections [4,6,19,25,29]. The structure of Piezo1, its activation mechanisms, cellular distribution within the osteoarthritic joint, and its physiological functions in cartilage homeostasis are summarized in Figure 3.

### 3.2. Cellular Expression of Piezo1 in the Osteoarthritic Joint

Piezo1 is widely expressed throughout the osteoarthritic joint, including articular chondrocytes, synovial fibroblasts, macrophages, osteoblasts, osteocytes, and other cells of the subchondral bone compartment. Under physiological conditions, Piezo1 contributes to tissue mechanoadaptation by coordinating cellular responses to mechanical stimulation and maintaining joint homeostasis. In OA, Piezo1 expression is increased in several joint compartments, particularly in mechanically overloaded cartilage, where enhanced channel activity promotes Ca^2+^ influx, inflammatory signaling, extracellular matrix degradation, and progressive tissue remodeling.

Importantly, Piezo1-mediated mechanotransduction is not restricted to articular cartilage. In the synovium, Piezo1 signaling in fibroblast-like synoviocytes and macrophages may translate mechanical stress and changes in the local biomechanical environment into Ca^2+^-dependent inflammatory responses. In particular, Piezo1 activation in synovial macrophages has been associated with pro-inflammatory polarization and activation of mitochondrial and innate immune signaling pathways. This response may provide a mechanism linking mechanical stress to persistent synovitis and cartilage catabolism.

Piezo1 also participates in mechanosensing within subchondral bone. Osteoblast-lineage cells and osteocytes respond to mechanical loading through mechanosensitive signaling pathways that regulate bone formation, remodeling, and adaptation to changes in joint loading. Dysregulation of these processes may contribute to abnormal subchondral bone remodeling and altered bone–cartilage mechanical crosstalk during OA progression. However, compared with articular chondrocytes, direct evidence linking Piezo1 dysregulation in human OA synovium and subchondral bone to disease progression remains comparatively limited. Collectively, these observations support a whole-joint perspective in which Piezo1 may coordinate mechanically induced responses across cartilage, synovium, immune cells, and subchondral bone rather than functioning exclusively as a cartilage mechanosensor [3,5,8,11].

Piezo2 also contributes to musculoskeletal mechanosensation and OA pathophysiology. However, the present review focuses on Piezo1 because the available evidence more consistently links Piezo1 to chondrocyte mechanotransduction, Ca^2+^ dysregulation, mitochondrial dysfunction, inflammatory signaling, and cartilage degeneration. Recent evidence indicates that chondrocyte Piezo1 and Piezo2 have overlapping but distinct functions in experimental OA, with Piezo2 deletion reducing pain and preserving activity without independently preventing cartilage degeneration [17]. Piezo2 also plays an important role in mechanical nociception, as Piezo2-expressing nociceptors contribute to mechanical sensitization in experimental OA [18]. Thus, the emphasis on Piezo1 reflects its closer mechanistic relationship with the mitochondrial and immunometabolic pathways that constitute the central focus of this review, rather than excluding the contribution of Piezo2 to OA pathophysiology.

### 3.3. Physiological Functions of Piezo1 in Cartilage Homeostasis

Physiological Piezo1 activation is essential for maintaining cartilage homeostasis by coordinating mechanosensing, calcium signaling, extracellular matrix turnover, and cellular adaptation to mechanical loading. Transient activation enables chondrocytes to respond appropriately to physiological forces, preserving tissue integrity and metabolic balance. In contrast, persistent activation induced by mechanical overload disrupts these adaptive mechanisms, resulting in sustained calcium influx and initiation of downstream catabolic pathways. These observations indicate that the biological effects of Piezo1 are highly context-dependent, with transient activation supporting tissue homeostasis and chronic activation promoting OA progression [4,10,14].

## 4. Mechanical Overload Initiates Piezo1 Signalling

Mechanical loading is indispensable for maintaining normal cartilage physiology. During daily activities, articular cartilage is continuously exposed to compressive, tensile, and shear forces that regulate ECM turnover and preserve tissue integrity. Under physiological conditions, these mechanical stimuli are tightly controlled and promote anabolic responses that maintain cartilage homeostasis. However, excessive or repetitive loading resulting from obesity, aging, traumatic injury, joint instability, malalignment, or abnormal gait profoundly alters the biomechanical microenvironment of chondrocytes. Instead of promoting tissue adaptation, pathological mechanical stress activates mechanosensitive signalling pathways that initiate progressive cartilage degeneration. Piezo1 is now widely recognized as a key mechanosensitive ion channel involved in converting mechanical stimuli into intracellular calcium signaling in articular chondrocytes. By sensing compressive, tensile, and shear forces, Piezo1 orchestrates early mechanotransduction events that maintain cartilage homeostasis under physiological loading but promote pathological signaling under chronic mechanical stress [4,5,7,10,25,27].

### 4.1. Mechanical Loading

Healthy cartilage exhibits remarkable biomechanical adaptability, allowing chondrocytes to sense and respond to dynamic alterations in joint loading. Moderate cyclic compression stimulates extracellular matrix synthesis and maintains collagen type II and aggrecan production, whereas excessive mechanical loading produces persistent Piezo1 activation and sustained intracellular calcium influx. Several recent investigations reported that mechanical overload significantly increases Piezo1 expression in osteoarthritic cartilage, thereby amplifying mechanotransduction and promoting catabolic signalling. Experimental stimulation using compressive stress or the Piezo1 agonist Yoda1 reproduces many pathological features observed in OA, whereas pharmacological inhibition of Piezo1 attenuates these responses, confirming that Piezo1 is not merely a mechanosensor but a critical molecular amplifier of mechanical injury [3,5,19,26,30,31,32].

Mechanical loading also modifies cytoskeletal organization and plasma membrane tension, facilitating channel opening through mechanical deformation of the lipid bilayer. This process initiates a cascade of intracellular events involving calcium-dependent activation of MAPK, ERK1/2, p38 MAPK, JNK, NF-κB and YAP/TAZ pathways, which collectively regulate inflammatory activation, extracellular matrix turnover and chondrocyte survival [13,14].

### 4.2. Extracellular Matrix Stiffness

The extracellular matrix is not only a structural scaffold but also an essential regulator of mechanobiological signalling. During OA progression, degradation of collagen fibrils, loss of proteoglycans and abnormal mineralization progressively increase cartilage stiffness while simultaneously reducing its ability to dissipate mechanical energy. These alterations expose resident chondrocytes to greater mechanical strain, establishing a positive feedback loop that further enhances Piezo1 activation.

Matrix stiffening increases membrane tension and lowers the activation threshold of Piezo1 channels, sensitizing chondrocytes to otherwise physiological mechanical stimuli. Consequently, progressively smaller mechanical forces become sufficient to induce pathological calcium influx, mitochondrial dysfunction and inflammatory signalling. This vicious cycle between extracellular matrix degradation and Piezo1 activation is increasingly recognized as a fundamental mechanism responsible for the transition from adaptive mechanobiology to irreversible cartilage degeneration [4,7,10].

### 4.3. Calcium Overload: The Central Trigger of Downstream Signalling

Intracellular calcium represents the principal second messenger linking Piezo1 activation to pathological cellular responses. While transient calcium oscillations regulate physiological chondrocyte metabolism, persistent Piezo1 activation is associated with sustained calcium overload that can substantially alter cellular homeostasis. Elevated cytosolic Ca^2+^ activates calmodulin-dependent kinases, calcineurin–nuclear factor of activated T cells (NFAT) signalling, mitogen-activated protein kinase (MAPK) pathways and NF-κB transcriptional activity, thereby promoting inflammatory gene expression and matrix degradation.

Excess intracellular calcium is rapidly transferred to mitochondria through the mitochondrial calcium uniporter, leading to mitochondrial calcium overload, excessive production of ROS, mitochondrial membrane depolarization and ATP depletion. These mitochondrial alterations further amplify oxidative stress and initiate mitophagy impairment, establishing the mechanistic link between mechanical overload and mitochondrial dysfunction discussed in the following sections of this review [8,9,10,11].

### 4.4. Piezo1 Drives Catabolic Signalling and Extracellular Matrix Degradation

Persistent activation of Piezo1 shifts cartilage metabolism from an anabolic toward a predominantly catabolic phenotype. Persistent Piezo1 activation markedly upregulates MMP-13 and ADAMTS5 while suppressing collagen II and aggrecan synthesis. Increased MMP-13 expression is accompanied by enhanced production of aggrecanases, particularly ADAMTS5, which cleaves aggrecan and disrupts the proteoglycan-rich extracellular matrix. Together, these enzymes represent the major mediators of cartilage matrix destruction in OA [5,10,32].

Simultaneously, Piezo1 activation suppresses the synthesis of essential anabolic extracellular matrix components, including collagen type II (COL2A1) and aggrecan (ACAN), thereby impairing cartilage repair and regeneration. Reduced expression of these structural proteins compromises cartilage biomechanical properties, further increasing tissue susceptibility to mechanical injury. Experimental inhibition or genetic silencing of Piezo1 consistently restores collagen II and aggrecan expression while reducing MMP-13 and ADAMTS5 levels, providing compelling evidence that Piezo1 occupies an upstream regulatory position controlling extracellular matrix homeostasis [5,10,20].

Collectively, current evidence supports a mechanistic model in which abnormal mechanical loading initiates Piezo1 activation, triggering pathological calcium influx and activation of multiple inflammatory and catabolic signalling pathways. The resulting imbalance between matrix synthesis and degradation progressively weakens the extracellular matrix, increases cartilage stiffness and further sensitizes Piezo1 to mechanical stimulation, thereby establishing a self-perpetuating feed-forward loop that drives OA progression. Importantly, this central position within the mechanobiological network identifies Piezo1 as an attractive upstream therapeutic target capable of simultaneously modulating mechanotransduction, calcium signalling, mitochondrial dysfunction and cartilage matrix preservation.

## 5. Piezo1-Induced Mitochondrial Dysfunction

**Figure 4:**
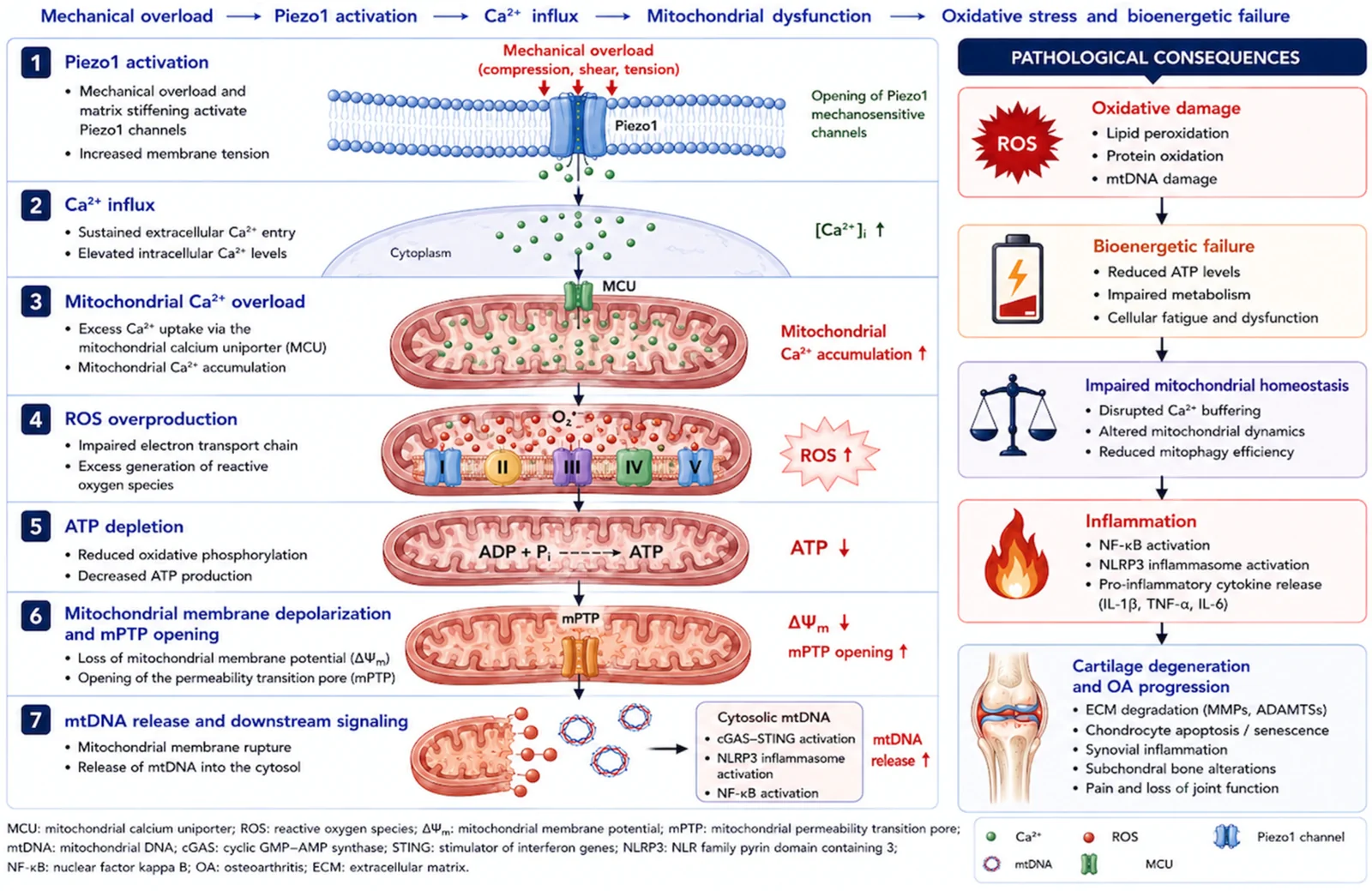
Piezo1-induced mitochondrial dysfunction in chondrocytes. The proposed sequence linking sustained mechanical overload to mitochondrial dysfunction is illustrated in seven steps: (1) mechanical overload and increased membrane tension activate Piezo1; (2) sustained Piezo1 opening increases intracellular Ca^2+^; (3) excessive mitochondrial Ca^2+^ uptake through the mitochondrial calcium uniporter (MCU) promotes mitochondrial Ca^2+^ overload; (4) disruption of mitochondrial electron transport increases reactive oxygen species (ROS) production; (5) impaired oxidative phosphorylation reduces adenosine triphosphate (ATP) production; (6) mitochondrial membrane depolarization and mitochondrial permeability transition pore (mPTP) opening compromise mitochondrial integrity; and (7) mitochondrial damage promotes mitochondrial DNA (mtDNA) release and downstream inflammatory signaling. These events may contribute to oxidative damage, bioenergetic failure, impaired mitochondrial homeostasis, inflammation, and ultimately cartilage degeneration and OA progression. Roman numerals I–V denote mitochondrial respiratory chain complexes I–V involved in oxidative phosphorylation. Abbreviations: ADAMTSs, a disintegrin and metalloproteinase with thrombospondin motifs; ATP, adenosine triphosphate; Ca^2+^, calcium; cGAS, cyclic GMP–AMP synthase; ECM, extracellular matrix; IL-1β, interleukin-1 beta; IL-6, interleukin-6; MCU, mitochondrial calcium uniporter; MMPs, matrix metalloproteinases; mtDNA, mitochondrial DNA; mPTP, mitochondrial permeability transition pore; NF-κB, nuclear factor kappa B; NLRP3, NOD-like receptor family pyrin domain containing 3; OA, osteoarthritis; ROS, reactive oxygen species; STING, stimulator of interferon genes; TNF-α, tumor necrosis factor-alpha; ΔΨm, mitochondrial membrane potential.

Mitochondria are central regulators of cellular metabolism, energy production, calcium homeostasis, redox balance, and cell survival. In articular chondrocytes, maintenance of mitochondrial integrity is essential for preserving extracellular matrix synthesis and adapting cellular metabolism to continuous mechanical stimulation. Mitochondrial dysfunction represents one of the earliest pathogenic events during OA, preceding overt cartilage degeneration and amplifying inflammatory and catabolic responses. Studies suggest that Piezo1 occupies a pivotal upstream position in this process by coupling excessive mechanical loading to mitochondrial injury through sustained intracellular calcium influx. Consequently, mitochondrial dysfunction is now recognized as a key mechanistic bridge linking abnormal mechanotransduction with impaired mitophagy, immunometabolic dysregulation, and progressive cartilage degeneration [8,9,10,11]. The sequential mechanisms linking Piezo1 activation to mitochondrial dysfunction and its pathological consequences are illustrated in Figure 4.

### 5.1. Mitochondrial Calcium Overload

Under physiological conditions, transient activation of Piezo1 generates tightly regulated calcium oscillations that participate in normal mechanotransduction and cartilage homeostasis. However, chronic mechanical overload induces persistent Piezo1 opening, producing sustained cytosolic Ca^2+^ accumulation. Excess calcium is rapidly transferred from the cytoplasm into mitochondria through the MCU, where it profoundly alters mitochondrial function.

Although moderate mitochondrial calcium uptake stimulates oxidative phosphorylation and ATP synthesis, excessive calcium accumulation disrupts mitochondrial homeostasis by promoting opening of the mitochondrial permeability transition pore (mPTP), swelling of the mitochondrial matrix, cristae disorganization, and activation of calcium-dependent proteases. These structural alterations impair mitochondrial respiration and initiate irreversible bioenergetic failure. Recent experimental studies demonstrated that inhibition or silencing of Piezo1 markedly reduces mitochondrial calcium overload and preserves mitochondrial morphology following mechanical stress, highlighting the direct contribution of Piezo1 to mitochondrial injury [5,8,10,21,35,37,41].

### 5.2. Reactive Oxygen Species Generation

One of the earliest consequences of mitochondrial calcium overload is excessive production of ROS. Under physiological conditions, mitochondria continuously generate low concentrations of ROS that function as essential intracellular signaling molecules regulating proliferation, differentiation, and extracellular matrix remodeling. However, excessive calcium influx disrupts electron transport chain efficiency, increasing electron leakage from respiratory complexes I and III and promoting uncontrolled generation of mitochondrial superoxide and hydrogen peroxide.

In osteoarthritic chondrocytes, excessive ROS production initiates oxidative damage to mitochondrial proteins, membrane phospholipids, and mtDNA, further compromising mitochondrial function. Importantly, ROS also amplify Piezo1-dependent signaling through activation of MAPK, NF-κB, and NLRP3 inflammasome pathways, establishing a positive feedback loop that perpetuates inflammation and extracellular matrix degradation. Multiple studies included in this review consistently demonstrate that pharmacological inhibition of Piezo1 significantly reduces intracellular ROS accumulation following mechanical overload, confirming that Piezo1 functions upstream of oxidative stress during OA progression [8,9,11,12,38,40].

### 5.3. ATP Depletion and Bioenergetic Failure

The progressive deterioration of mitochondrial function ultimately compromises ATP production, the principal source of cellular energy required for extracellular matrix synthesis, ion transport, cytoskeletal organization, and tissue repair. Healthy chondrocytes depend predominantly on glycolysis because of the hypoxic cartilage microenvironment; nevertheless, mitochondrial oxidative phosphorylation remains essential for maintaining intracellular energy balance and supporting anabolic metabolism.

Persistent Piezo1 activation induces profound alterations in mitochondrial respiration, reducing oxidative phosphorylation efficiency while increasing proton leakage across the inner mitochondrial membrane. Consequently, ATP synthesis declines despite increased metabolic demand imposed by mechanical injury. Bioenergetic failure impairs collagen type II and aggrecan synthesis, suppresses matrix repair, and sensitizes chondrocytes to apoptosis and other forms of regulated cell death. Experimental studies have demonstrated that restoring mitochondrial function or inhibiting Piezo1 partially preserves ATP production and attenuates cartilage degeneration, emphasizing the importance of mitochondrial bioenergetics in OA pathogenesis [10,20,36,39,44,45].

### 5.4. Mitochondrial Membrane Depolarization

Maintenance of the mitochondrial membrane potential (ΔΨm) is essential for ATP generation, calcium buffering, protein import, and mitochondrial quality control. Excessive calcium influx and ROS accumulation progressively dissipate ΔΨm, resulting in mitochondrial membrane depolarization, impaired electron transport chain activity, and activation of mitochondrial stress responses.

Loss of ΔΨm represents one of the earliest indicators of mitochondrial dysfunction and frequently precedes apoptosis or mitophagy activation. In mechanically overloaded chondrocytes, Piezo1-mediated calcium overload accelerates mitochondrial depolarization, promoting cytochrome c release, activation of caspase-dependent apoptotic pathways, and increased susceptibility to cartilage degeneration. Furthermore, persistent depolarization impairs mitochondrial dynamics by disrupting the balance between fusion and fission, thereby facilitating fragmentation of the mitochondrial network and reducing mitochondrial resilience to mechanical stress [8,9,11,47,48,49].

However, mitochondrial depolarization should be interpreted cautiously. Commonly used ΔΨm-sensitive fluorescent probes provide indirect and context-dependent estimates of mitochondrial membrane potential and can be influenced by probe concentration, mitochondrial mass, loading conditions, and experimental design. Thus, changes in ΔΨm should ideally be interpreted together with complementary measures of mitochondrial function rather than considered in isolation.

### 5.5. Oxidative Stress: The Link Between Mitochondrial Dysfunction and Cartilage Degeneration

Oxidative stress represents the cumulative consequence of mitochondrial calcium overload, excessive ROS generation, ATP depletion, and mitochondrial membrane depolarization. When antioxidant defenses become overwhelmed, oxidative damage extends beyond mitochondria to affect nuclear DNA, extracellular matrix proteins, membrane lipids, and intracellular signaling molecules. This widespread molecular injury activates inflammatory pathways, accelerates extracellular matrix degradation, and promotes cellular senescence.

Several studies demonstrate that oxidative stress induced by Piezo1 activation markedly increases expression of MMP-13, ADAMTS5, inducible nitric oxide synthase (iNOS), cyclooxygenase-2 (COX-2), and multiple pro-inflammatory cytokines while simultaneously suppressing anabolic markers such as collagen type II and aggrecan. Importantly, mitochondrial oxidative stress also promotes release of mtDNA into the cytoplasm, thereby activating cGAS–STING signaling and the NLRP3 inflammasome, which further amplify immunometabolic dysregulation. These observations place mitochondrial dysfunction at the center of the pathological cascade linking abnormal mechanical loading to chronic inflammation and progressive cartilage destruction [8,12,13,14,22,23].

Together, current evidence supports a hierarchical model in which pathological mechanical loading activates Piezo1, initiating sustained calcium influx that drives mitochondrial calcium overload, ROS production, ATP depletion, membrane depolarization, and oxidative stress. Rather than representing isolated events, these alterations constitute an interconnected mitochondrial stress response that amplifies inflammation, extracellular matrix degradation, and chondrocyte dysfunction. Importantly, mitochondrial injury also activates adaptive quality-control mechanisms, particularly mitophagy, which initially attempts to preserve cellular homeostasis but progressively become insufficient during persistent mechanical stress. Consequently, impaired mitophagy emerges as the next critical step in Piezo1-mediated osteoarthritis progression and is discussed in the following section.

## 6. Piezo1-Associated Mitophagy: A Central Mechanism Linking Mechanotransduction to Osteoarthritis Progression

Mitophagy is a highly conserved mitochondrial quality-control mechanism responsible for the selective recognition and lysosomal degradation of dysfunctional mitochondria. By eliminating damaged mitochondria before irreversible cellular injury occurs, mitophagy maintains mitochondrial bioenergetics, limits excessive ROS production, preserves calcium homeostasis, and prevents activation of inflammatory pathways. In healthy articular cartilage, mitophagy represents an adaptive response that enables chondrocytes to tolerate repetitive mechanical loading while maintaining ECM homeostasis. However, studies suggest that persistent Piezo1 activation disrupts mitochondrial quality control, progressively overwhelming mitophagic capacity and promoting accumulation of dysfunctional mitochondria. Consequently, impaired mitophagy has emerged as a pivotal event linking mechanical overload to mitochondrial dysfunction, immunometabolic dysregulation, cellular senescence, and progressive cartilage degeneration [8,9,10].

### 6.1. Physiological Mitophagy: Maintaining Mitochondrial Homeostasis

Under physiological conditions, mitochondrial turnover is a continuous and tightly regulated process. Mechanical stimulation transiently increases mitochondrial calcium uptake and oxidative phosphorylation, leading to moderate ROS production that participates in intracellular signaling without inducing cellular injury. Damaged mitochondria are rapidly identified through alterations in mitochondrial membrane potential (ΔΨm) and removed by mitophagy before oxidative damage accumulates. This process preserves ATP production, limits oxidative stress, and maintains chondrocyte viability despite continuous biomechanical loading.

Physiological mitophagy also regulates mitochondrial dynamics by coordinating mitochondrial fusion and fission, thereby maintaining a healthy mitochondrial network capable of adapting to fluctuating metabolic demands. During OA progression, persistent mechanical overload mediated by Piezo1 progressively exceeds the capacity of these protective mechanisms, resulting in defective mitochondrial turnover and accumulation of dysfunctional mitochondria that amplify inflammatory signaling [9,11].

### 6.2. The PINK1/Parkin Pathway: The Canonical Mitophagy Machinery

Among the molecular pathways regulating mitophagy, the PTEN-induced kinase 1 (PINK1)/Parkin pathway represents the best-characterized mechanism responsible for eliminating depolarized mitochondria. Under physiological conditions, PINK1 is continuously imported into healthy mitochondria and rapidly degraded. Loss of mitochondrial membrane potential prevents PINK1 degradation, leading to its accumulation on the outer mitochondrial membrane where it recruits and activates the E3 ubiquitin ligase Parkin.

Activated Parkin ubiquitinates multiple mitochondrial outer membrane proteins, including mitofusins (Mfn1 and Mfn2), voltage-dependent anion channel (VDAC), and mitochondrial Rho GTPases, thereby promoting recruitment of autophagic adaptor proteins such as p62/SQSTM1, OPTN, and NDP52. Subsequent interaction with LC3-positive autophagosomes enables selective removal of dysfunctional mitochondria through lysosomal degradation. Recent evidence further supports this relationship, showing that enhancement of PINK1/Parkin-mediated mitophagy can restore mitochondrial homeostasis and limit NLRP3-associated inflammatory cell death in OA chondrocytes [16].

Current evidence suggests that sustained Piezo1-associated Ca^2+^ influx and mitochondrial stress may contribute to alterations in PINK1/Parkin-dependent mitophagy. However, the available evidence remains largely indirect, and a direct causal relationship between Piezo1 activation and PINK1/Parkin dysfunction has not yet been established, particularly in human OA chondrocytes. Such impaired mitochondrial quality control may promote the accumulation of dysfunctional mitochondria, thereby contributing to sustained ROS production, ATP depletion, mtDNA release, and chronic inflammatory activation [8,9,11].

### 6.3. BNIP3-Mediated Receptor-Dependent Mitophagy

In addition to the ubiquitin-dependent PINK1/Parkin pathway, receptor-mediated mitophagy represents an important complementary mechanism regulating mitochondrial turnover. BCL2/adenovirus E1B 19-kDa interacting protein 3 (BNIP3) is an outer mitochondrial membrane receptor containing a conserved LC3-interacting region (LIR) that directly recruits autophagosomes independently of Parkin. Consistent with this concept, recent preclinical evidence indicates that activation of the AMPK/PINK1/Parkin axis can enhance mitophagy and attenuate inflammatory and matrix-degrading responses in experimental OA models [16].

BNIP3-mediated mitophagy is particularly important under conditions of hypoxia and oxidative stress, both characteristic features of osteoarthritic cartilage. Because articular cartilage is naturally avascular, chondrocytes rely on adaptive mitochondrial responses to maintain energy production under limited oxygen availability. Chronic Piezo1 activation disrupts this adaptive process by increasing mitochondrial calcium overload and oxidative injury, potentially impairing BNIP3-mediated mitochondrial clearance. Although direct evidence remains limited, emerging studies suggest that altered BNIP3 expression contributes to defective mitochondrial quality control during OA progression [9,11].

### 6.4. FUNDC1: Integrating Mechanical Stress with Mitophagy

FUN14 domain-containing protein 1 (FUNDC1) represents another hypoxia-responsive mitophagy receptor located on the outer mitochondrial membrane. Similar to BNIP3, FUNDC1 directly interacts with LC3 through its LIR motif, promoting selective elimination of dysfunctional mitochondria independently of ubiquitination.

Recent evidence suggests that FUNDC1 is highly sensitive to alterations in mitochondrial membrane potential, calcium homeostasis, and ROS generation, all of which are profoundly influenced by Piezo1 activation. Persistent mechanical overload may therefore impair FUNDC1-dependent mitochondrial quality control, further amplifying mitochondrial dysfunction and inflammatory activation. Although studies directly investigating the Piezo1–FUNDC1 axis remain scarce, this signaling pathway represents an important area for future investigation because it may connect mechanotransduction with hypoxia-dependent mitochondrial adaptation.

### 6.5. DRP1: Mitochondrial Fission as a Prerequisite for Mitophagy

Mitochondrial fission constitutes an essential preparatory step preceding mitophagy. Dynamin-related protein 1 (DRP1) is recruited from the cytoplasm to the mitochondrial outer membrane, where it constricts and divides damaged mitochondria into smaller fragments suitable for autophagic degradation.

Studies have identified DRP1 as a critical downstream effector of Piezo1 signaling. Excessive calcium influx activates calcineurin-dependent DRP1 dephosphorylation, promoting excessive mitochondrial fragmentation. While physiological fission facilitates efficient mitochondrial quality control, uncontrolled DRP1 activation results in excessive mitochondrial fragmentation, bioenergetic failure, ROS overproduction, and activation of inflammatory pathways, including cGAS–STING and the NLRP3 inflammasome. Notably, a recent study reported that Piezo1 activation in synovial macrophages was associated with DRP1-dependent mitochondrial dysfunction, cGAS–STING signaling, and accelerated osteoarthritis progression [8]. These findings support a potential role for DRP1 at the intersection of mechanotransduction, mitochondrial dynamics, innate immunity, and cartilage degeneration.

### 6.6. Mitochondrial Fusion and Fission: Balancing Mfn1, Mfn2 and OPA1

Maintenance of a functional mitochondrial network depends on the dynamic balance between mitochondrial fusion and fission. Fusion enables functional complementation between damaged and healthy mitochondria, preserving ATP production and limiting oxidative stress, whereas fission isolates dysfunctional mitochondria for subsequent mitophagic removal.

Mitofusin 1 (Mfn1) and Mitofusin 2 (Mfn2) regulate fusion of the outer mitochondrial membrane, whereas optic atrophy protein 1 (OPA1) controls fusion of the inner mitochondrial membrane and maintains cristae architecture. In healthy cartilage, coordinated activity of Mfn1, Mfn2 and OPA1 preserves mitochondrial morphology and optimizes oxidative phosphorylation. During OA progression, persistent Piezo1 activation disrupts this equilibrium by promoting excessive DRP1-dependent fission while simultaneously reducing mitochondrial fusion, resulting in fragmented mitochondria characterized by reduced ATP production, increased ROS generation, membrane depolarization and defective mitophagy.

**Figure 5:**
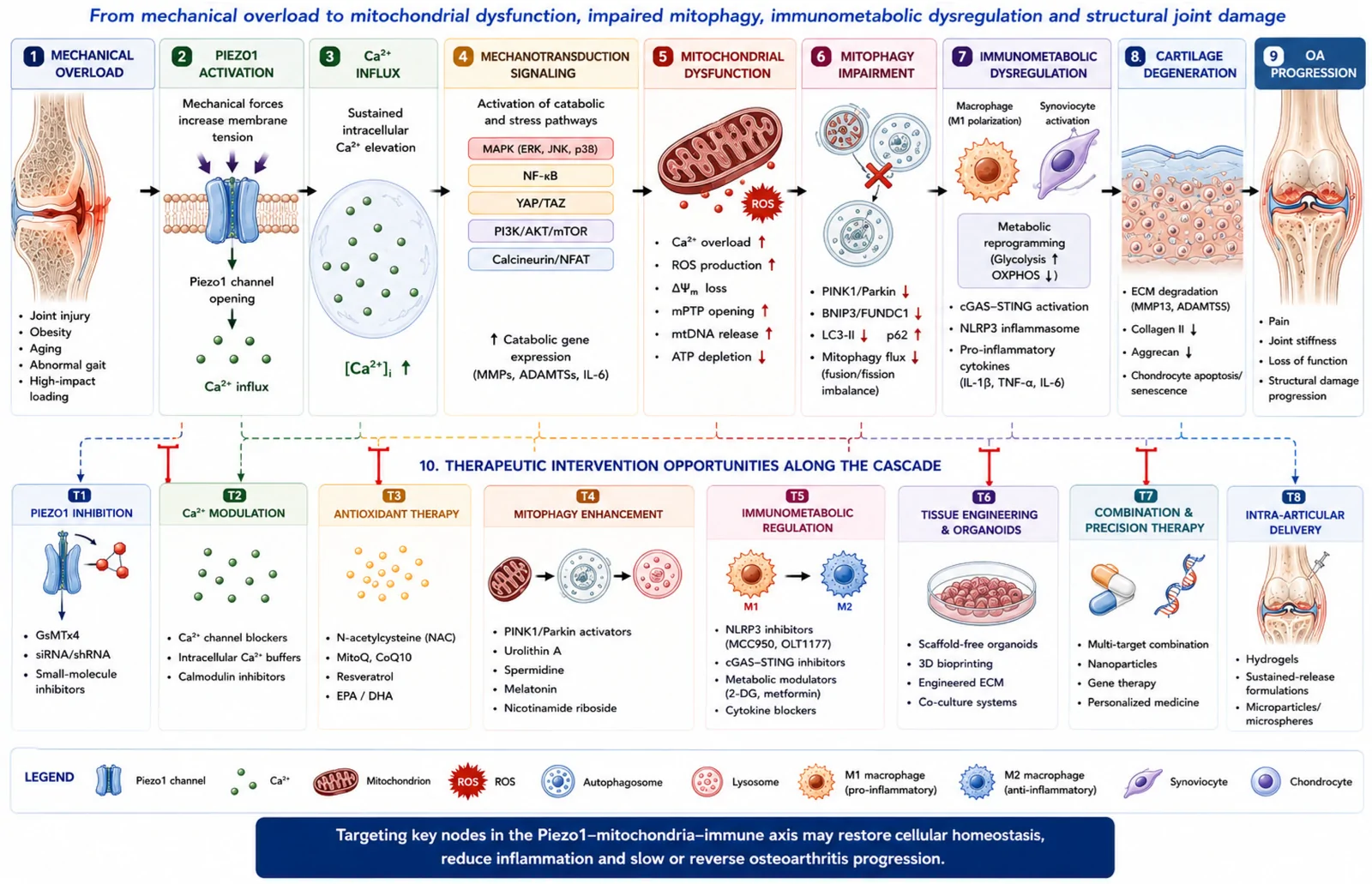
Piezo1-mediated immunometabolic cascade linking mechanical overload to osteoarthritis progression and potential therapeutic intervention points. The proposed cascade integrates the principal mechanisms linking pathological mechanical loading to OA progression: (1) mechanical overload activates Piezo1; (2) sustained Ca^2+^ influx promotes intracellular calcium overload; (3) mitochondrial Ca^2+^ accumulation contributes to mitochondrial dysfunction, increased ROS production, ATP depletion, mitochondrial membrane depolarization, and mtDNA release; (4) mitochondrial quality-control mechanisms and mitophagy become dysregulated; (5) mitochondrial damage and mtDNA release promote activation of innate immune pathways, including cGAS–STING and the NLRP3 inflammasome; (6) inflammatory signaling promotes macrophage polarization and cytokine production; (7) persistent inflammation enhances extracellular matrix catabolism; (8) cartilage degeneration and alterations in the joint microenvironment further modify mechanical loading; and (9) these interconnected processes may establish a self-amplifying mechanobiological and immunometabolic cycle contributing to OA progression. Potential therapeutic intervention points (T1–T8) are positioned along the cascade, ranging from modulation of Piezo1 activity and intracellular Ca^2+^ signaling to mitochondrial protection, restoration of mitochondrial quality control, inhibition of innate immune and inflammatory pathways, and cartilage-protective strategies. Abbreviations: ADAMTS5, a disintegrin and metalloproteinase with thrombospondin motifs 5; AKT, protein kinase B; ATP, adenosine triphosphate; BNIP3, BCL2 interacting protein 3; Ca^2+^, calcium; cGAS, cyclic GMP–AMP synthase; DRP1, dynamin-related protein 1; ECM, extracellular matrix; ERK, extracellular signal-regulated kinase; FUNDC1, FUN14 domain-containing protein 1; GsMTx4, Grammostola spatulata mechanotoxin 4; IL-1β, interleukin-1 beta; IL-6, interleukin-6; JNK, c-Jun N-terminal kinase; LC3-II, microtubule-associated protein 1 light chain 3-II; MAPK, mitogen-activated protein kinase; MMP-13, matrix metalloproteinase 13; mtDNA, mitochondrial DNA; mPTP, mitochondrial permeability transition pore; mTOR, mechanistic target of rapamycin; NF-κB, nuclear factor kappa B; NLRP3, NOD-like receptor family pyrin domain containing 3; OA, osteoarthritis; OXPHOS, oxidative phosphorylation; p62, sequestosome 1; PI3K, phosphoinositide 3-kinase; PINK1, PTEN-induced kinase 1; ROS, reactive oxygen species; siRNA, small interfering RNA; STING, stimulator of interferon genes; TNF-α, tumor necrosis factor-alpha; YAP/TAZ, yes-associated protein/transcriptional coactivator with PDZ-binding motif; ΔΨm, mitochondrial membrane potential.

Accumulating evidence therefore supports the concept that altered mitochondrial dynamics represent a central pathogenic event downstream of Piezo1 activation. Restoring the balance between mitochondrial fusion and fission may preserve mitochondrial integrity, improve mitophagic efficiency and reduce inflammatory activation, making mitochondrial dynamics an attractive therapeutic target in OA [8,9,10,11]. Figure 5 provides an integrated overview of the mechanistic pathways linking mechanical overload, Piezo1 activation, mitochondrial dysfunction, impaired mitophagy, immunometabolic dysregulation, and osteoarthritis progression.

## 7. Therapeutic Opportunities Targeting the Piezo1–Mitochondria Axis

**Figure 6:**
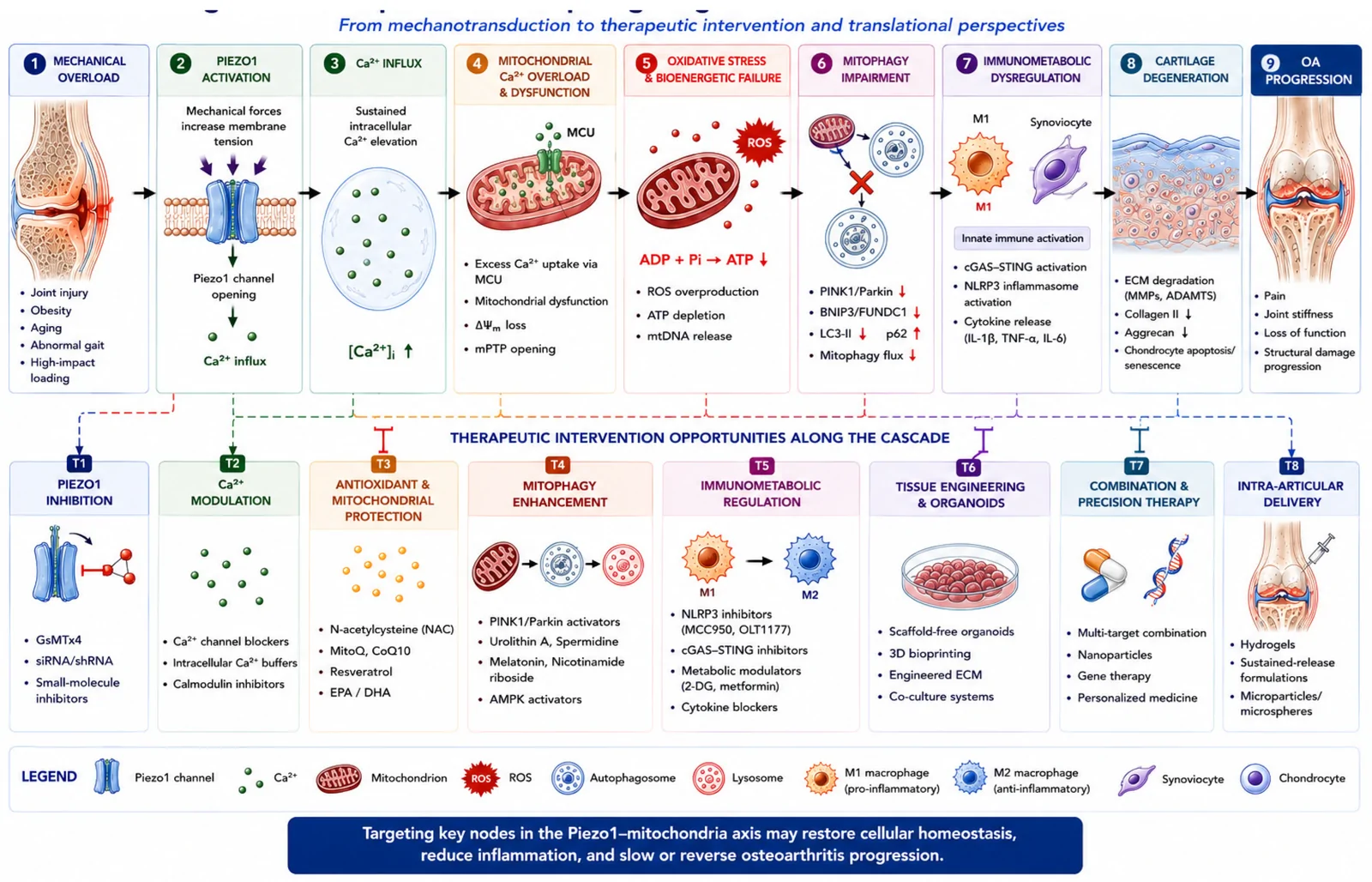
Therapeutic roadmap targeting the Piezo1–mitochondria axis in osteoarthritis. The figure summarizes current and emerging therapeutic strategies aimed at modulating different components of the Piezo1–mitochondria–immune axis. These approaches include direct or indirect modulation of Piezo1 activity, regulation of Ca^2+^ homeostasis, mitochondrial protection and antioxidant strategies, enhancement of mitophagy and mitochondrial quality control, modulation of mitochondrial dynamics and metabolic pathways, inhibition of innate immune and inflammatory signaling, and cartilage-protective interventions. Local and targeted delivery approaches, including RNA-based therapies, biomaterials, and nanomedicine, may improve tissue specificity and reduce systemic exposure. However, most of these strategies remain experimental or preclinical, and their efficacy, specificity, delivery efficiency, pharmacokinetics, and long-term safety require further validation before clinical translation in human OA. Abbreviations: 2-DG, 2-deoxy-D-glucose; ADAMTS, a disintegrin and metalloproteinase with thrombospondin motifs; ADP, adenosine diphosphate; AMPK, AMP-activated protein kinase; ATP, adenosine triphosphate; BNIP3, BCL2 interacting protein 3; Ca^2+^, calcium; cGAS, cyclic GMP–AMP synthase; CoQ10, coenzyme Q10; DHA, docosahexaenoic acid; ECM, extracellular matrix; EPA, eicosapentaenoic acid; FUNDC1, FUN14 domain-containing protein 1; GsMTx4, Grammostola spatulata mechanotoxin 4; IL-1β, interleukin-1 beta; IL-6, interleukin-6; LC3-II, microtubule-associated protein 1 light chain 3-II; MCU, mitochondrial calcium uniporter; MMPs, matrix metalloproteinases; mtDNA, mitochondrial DNA; NAC, N-acetylcysteine; NLRP3, NOD-like receptor family pyrin domain containing 3; OA, osteoarthritis; p62, sequestosome 1; Pi, inorganic phosphate; PINK1, PTEN-induced kinase 1; ROS, reactive oxygen species; shRNA, short hairpin RNA; siRNA, small interfering RNA; STING, stimulator of interferon genes; TNF-α, tumor necrosis factor-alpha.

The identification of Piezo1 as a central mechanosensor linking abnormal mechanical loading to mitochondrial dysfunction has opened new opportunities for disease-modifying therapies in OA. Rather than exclusively alleviating pain, emerging strategies aim to interrupt the mechanobiological cascade connecting Piezo1 activation to mitochondrial damage, impaired mitophagy, inflammation, and extracellular matrix degradation. Current approaches target either Piezo1 directly or its downstream mitochondrial and inflammatory pathways, while advanced delivery systems seek to improve therapeutic specificity and reduce systemic adverse effects. Current and emerging therapeutic strategies targeting the Piezo1–mitochondria axis are summarized in Table 3 and illustrated in Figure 6.

### 7.1. Modulation of Piezo1 Activity

Direct modulation of Piezo1 represents the most upstream therapeutic strategy. Genetic deletion or silencing of Piezo1 consistently reduces calcium overload, chondrocyte apoptosis, inflammatory signaling, and cartilage degeneration in experimental OA models [24,42,52]. However, accumulating evidence indicates that Piezo1 also contributes to physiological cartilage mechanoadaptation and regeneration. For example, transient Piezo1 activation promoted Procr-positive chondroprogenitor-mediated cartilage repair, whereas complete inhibition impaired regenerative responses [14]. These observations suggest that therapeutic strategies should aim to normalize rather than completely suppress Piezo1 activity.

Pharmacological modulation has mainly relied on GsMTx4, a mechanosensitive channel inhibitor that attenuates calcium influx, apoptosis, ferroptosis, and extracellular matrix degradation while preserving collagen II and aggrecan expression [34,43]. Conversely, the Piezo1 agonist Yoda1 has been invaluable for elucidating Piezo1 biology but primarily reproduces pathological mechanical loading. Although its effects appear context-dependent, Yoda1 remains principally an experimental tool rather than a therapeutic candidate.

### 7.2. Restoring Mitochondrial Homeostasis

Because mitochondrial dysfunction represents the major downstream consequence of Piezo1 activation, therapies restoring mitochondrial homeostasis constitute an attractive complementary approach. Omega-3 polyunsaturated fatty acids, particularly eicosapentaenoic acid (EPA), reduce Piezo1 activation by modifying plasma membrane properties, thereby limiting calcium overload, oxidative stress, and chondrocyte apoptosis [3,4].

Similarly, mitochondrial-targeted antioxidants—including MitoQ, coenzyme Q10, and glutathione-dependent pathways—attenuate ROS production and ferroptosis associated with Piezo1 activation [43]. Restoration of mitochondrial quality control through enhancement of PINK1/Parkin-dependent mitophagy also represents a promising strategy. Although direct evidence remains limited, activation of mitophagy and modulation of the PI3K–AKT–mTOR pathway have shown encouraging experimental results in preserving mitochondrial integrity and reducing cartilage degeneration [13,51].

### 7.3. Advanced Therapeutic Strategies

Emerging precision therapies seek to selectively target Piezo1 within the osteoarthritic joint. RNA-based approaches, including siRNA and gene silencing, have successfully reduced Piezo1 expression and improved cartilage preservation in experimental models [11]. Particularly promising are mannose-modified lipid nanoparticles delivering Piezo1 siRNA specifically to synovial macrophages, thereby suppressing the DRP1–cGAS–STING–NLRP3 inflammatory axis and slowing OA progression [8].

Hydrogels and nanomedicine further enhance local drug delivery by increasing retention within the joint while minimizing systemic exposure. These biomaterial-based platforms may simultaneously provide mechanical support and sustained release of Piezo1 inhibitors, antioxidants, or mitophagy modulators, representing an important step toward targeted disease-modifying therapies [46].

Overall, the proposed Piezo1–mitochondria–mitophagy axis should therefore be regarded as an emerging mechanistic framework rather than a fully established causal pathway in human OA. Most supporting evidence derives from cell-based experiments and preclinical animal models, with differences in mechanical loading paradigms, OA induction methods, cell sources, and experimental endpoints contributing to heterogeneity across studies. Direct validation in human OA tissues and longitudinal or interventional studies remains limited. These considerations are particularly important when translating mechanistic observations into therapeutic strategies targeting Piezo1 or mitochondrial quality-control pathways.

### 7.4. Future Therapeutic Perspectives

Given the multifaceted role of Piezo1 in cartilage biology, a single-target strategy is unlikely to provide optimal clinical benefit. Future therapies will probably combine moderate Piezo1 modulation with mitochondrial protection, restoration of mitophagy, and inhibition of downstream inflammatory pathways. Such approaches should be guided by biomarkers reflecting Piezo1 activity, mitochondrial dysfunction, and inflammatory status to enable patient stratification and precision medicine. Together, these advances position the Piezo1–mitochondria axis as one of the most promising therapeutic targets for the development of disease-modifying osteoarthritis therapies.

Despite the therapeutic potential of Piezo1 modulation, clinical translation remains at an early stage. Most available evidence derives from cell-based experiments and preclinical OA models, and direct pharmacological targeting of Piezo1 has not yet been clinically validated in OA. GsMTx4 has shown chondroprotective effects in experimental models by attenuating Piezo1-associated Ca^2+^ influx, chondrocyte apoptosis, ferroptosis, and extracellular matrix degradation [20,36]. However, these findings remain preclinical and the specificity, pharmacokinetic properties, delivery, and long-term safety of Piezo1-directed channel modulation require further investigation. RNA-based strategies also appear promising: Piezo1 silencing has improved cartilage-related outcomes in experimental OA models, while macrophage-targeted delivery of Piezo1 siRNA using mannose-modified lipid nanoparticles reduced M1 polarization and inhibited the DRP1–cGAS–STING–NLRP3 inflammatory axis in vivo [8]. Artemisinin has also been reported to inhibit Piezo1 activation and attenuate OA lesions in experimental models, supporting the potential for pharmacological modulation of Piezo1 [42]. Nevertheless, these approaches remain investigational, and their specificity, delivery efficiency, pharmacokinetics, and long-term safety must be established before clinical translation. Membrane-directed approaches such as EPA may provide an alternative strategy by reducing Piezo mechanosensitivity rather than completely blocking channel activity [3,4]. Overall, current evidence favors local, targeted, or context-dependent modulation of Piezo1 over indiscriminate systemic inhibition, but robust human interventional data are still lacking.

## 8. Conclusions

Piezo1 has emerged as a key mechanosensitive ion channel linking abnormal mechanical loading to mitochondrial dysfunction, impaired mitophagy, immunometabolic dysregulation, and progressive cartilage degeneration in osteoarthritis. Beyond mediating mechanically induced ion flux, Piezo1 influences calcium signaling, mitochondrial quality control, inflammatory activation, and extracellular matrix remodeling, supporting an important role in OA pathogenesis.

Current evidence indicates that persistent Piezo1 activation induces mitochondrial calcium overload, excessive ROS production, ATP depletion, and mitochondrial membrane depolarization, ultimately triggering defective mitophagy and activation of the cGAS–STING and NLRP3 inflammatory pathways. These events may contribute to a self-amplifying cycle that promotes chondrocyte dysfunction, synovial inflammation, and cartilage destruction.

Importantly, Piezo1 also exerts physiological functions in cartilage mechanoadaptation and tissue homeostasis, indicating that future therapeutic strategies should aim to normalize rather than completely inhibit its activity. Emerging technologies, including scaffold-free organoids, single-cell and spatial transcriptomics, artificial intelligence, and targeted drug-delivery systems, provide unprecedented opportunities to decipher Piezo1 biology and accelerate translational research.

Although significant challenges remain, targeting the Piezo1–mitochondria axis represents a promising strategy for developing disease-modifying therapies. Future studies integrating mechanobiology, mitochondrial medicine, immunology, and tissue engineering will be essential to translate these experimental discoveries into personalized treatments capable of slowing or preventing osteoarthritis progression.

## Figures and Tables

**Table 1:** Distribution of the 47 Included Studies According to the Main Themes of the Review.

Manuscript Section	Scientific Focus	Core Studies (Reference Number)	Supporting Studies (Reference Number)
Piezo1: A Key Mechanosensitive Ion Channel in Cartilage	Piezo1 biology, mechanotransduction, calcium signalling and cartilage homeostasis	(Xiaohui Wang et al. 2024) [5]; (Jurynec et al. 2026) [25]; (Y. Zhang et al. 2026) [26]; (Steinecker-Frohnwieser et al. 2023) [27]; (Feng et al. 2024) [28]	(Marushack et al. 2025) [4]; (Z. Yan et al. 2026) [8]; (Savadipour et al. 2026) [29]
Mechanical Overload Initiates Piezo1 Signalling	Mechanical loading, extracellular matrix stiffness, calcium overload and catabolic signalling	(F. Chen et al. 2024) [3]; (X. Yan et al. 2025) [7]; (S. Chen et al. 2026) [10]; (F. Liu et al. 2026) [30]; (Savadipour et al. 2023) [31]; (Zhuang et al. 2023) [32]	(Lee et al. 2021) [33]; (Ren et al. 2023) [34]
Piezo1-Induced Mitochondrial Dysfunction	Mitochondrial calcium overload, ROS production, ATP depletion, membrane depolarization and oxidative stress	(X. Yan et al. 2025) [7]; (Zhu et al. 2025) [14]; (Yang et al. 2025) [35]; (Xinyu Wang et al. 2025) [36]; (J. Du et al. 2025) [37]; (L. Sun et al. 2025) [38]; (Y. Zhou et al. 2025) [39]; (Ren et al. 2022) [40]; (Yi Sun et al. 2021) [41]; (Gan et al. 2024) [42]	(Z. Yan et al. 2026) [8]; (Yu et al. 2025) [13]; (Steinecker-Frohnwieser et al. 2023) [27]; (Savadipour et al. 2023) [31]; (Wang et al. 2022) [43]; (S. Han 2022) [44]; (Hu et al. 2023) [45]; (Y. Zhang et al. 2026) [46]
Piezo1-Associated Mitophagy	PINK1/Parkin signalling, mitochondrial quality control, mitochondrial dynamics and immunometabolic regulation	(X. Yan et al. 2025) [7]; (Yu et al. 2025) [13]; (Zhu et al. 2025) [14]; (Yang et al. 2025) [35]; (Xinyu Wang et al. 2025) [36]; (L. Sun et al. 2025) [38]; (Y. Zhou et al. 2025) [39]; (L.-Z. Zhang et al. 2026) [47]; (Liang et al. 2026) [48]; (Z. Liu et al. 2026) [49]	(Savadipour et al. 2023) [31]; (Zhuang et al. 2023) [32]; (Ren et al. 2022) [40]; (Yi Sun et al. 2021) [41]
Therapeutic Opportunities Targeting the Piezo1–Mitochondria Axis	Piezo1 inhibitors, mitochondrial protection, mitophagy activation, biomaterials and targeted delivery	(Gan et al. 2024) [42]; (Y. Zhang et al. 2026) [46]; (M. Liu et al. 2025) [50]; (Y. Han et al. 2025) [51]; (Kan et al. 2026) [52]	(Marushack et al. 2025) [4]; (X. Yan et al. 2025) [7]; (Lee et al. 2021) [33]; (Ren, Zhuang, Li, et al. 2023) [34]; (Palmer et al. 2025) [53]; (J. Li et al. 2023) [54]; (G. Du et al. 2022) [55]; (C.-B. Wu et al. 2022) [56]; (Shao et al. 2026) [57]
Other Relevant Topics	Transcriptomics, multi-omics, bioinformatics, disease biology and emerging concepts related to osteoarthritis	(H. Zhang et al. 2025) [58]; (L.-L. Li et al. 2021) [59]	(Yuting Sun et al. 2023) [60]; (Ren, Zhuang, Jiang, et al. 2023) [61]; (Batarfi et al. 2025) [62]; (Selig et al. 2020) [63]

**Table 2:** Representative Experimental Models and Levels of Evidence Supporting the Piezo1–Mitochondria–Immune Axis in Osteoarthritis. Representative studies are summarized by model, biological source, intervention/exposure, principal findings, and evidence category. Categories are descriptive rather than a formal clinical grading system.

Study	Experimental Model	Species/Biological Source	Intervention/Exposure	Major Findings	Evidence Category
(Lee et al. 2021) [33]	Primary chondrocyte culture + human OA cartilage	Porcine chondrocytes; human OA cartilage	IL-1α exposure; mechanical deformation; Piezo1 pathway analysis	Inflammatory signaling increased Piezo1 expression and Ca^2+^ mechanosensitivity, creating a feed-forward mechanism that amplified mechanical injury.	Human tissue + in vitro mechanistic
(Steinecker-Frohnwieser et al. 2023) [27]	Primary human chondrocyte culture	Healthy and OA human articular chondrocytes	Piezo1/TRPV4 activation and channel-interaction studies	Demonstrated Piezo1–TRPV4 crosstalk and modulation of OA-relevant gene expression in primary human chondrocytes.	Human in vitro mechanistic
(Savadipour et al. 2023) [31]	Cell-based mechanotransduction model	Articular chondrocytes	Controlled membrane stretch/mechanical stimulation	Supported membrane stretch as a direct mechanism of PIEZO1 activation in chondrocytes.	In vitro mechanistic
(Ren et al. 2023) [34]	Mechanically overloaded chondrocytes + experimental OA	Chondrocytes; preclinical OA model	Excessive mechanical strain; GsMTx4 inhibition	GsMTx4 attenuated Piezo1–calcineurin–NFAT1 signaling, apoptosis, and cartilage catabolism under excessive mechanical strain.	Multi-model preclinical
(Wang et al. 2022) [43]	Mechanical-overload/ferroptosis model	Chondrocytes; experimental OA model	Mechanical overloading; Piezo1 blockade/GPX4 pathway analysis	Piezo1-facilitated Ca^2+^ influx promoted GPX4-regulated ferroptosis; channel blockade reduced ferroptotic and OA-related changes.	Multi-model preclinical
(Gan et al. 2024) [42]	Cell-based + animal OA therapeutic model	Chondrocytes; experimental OA model	Piezo1 activation; artemisinin treatment	Piezo1 activation accelerated OA-related changes, whereas artemisinin reduced Piezo1 activity and attenuated OA lesions.	Multi-model preclinical
(Jia et al., 2024) [11]	In vivo gene-silencing OA model	Rat OA model	siRNA double silencing of Piezo1 and TRPV4	Gene silencing improved cartilage-related outcomes in experimental OA, supporting RNA-based mechanosensitive-channel targeting.	In vivo preclinical
(Zhu et al., 2025) [14]	Chondroprogenitor/cartilage-regeneration model	Procr-positive chondroprogenitors; experimental OA/cartilage model	Manipulation of Piezo1 activity during mechanical stimulation	Showed context-dependent Piezo1 biology: transient activation supported chondroprogenitor-mediated cartilage maintenance and regeneration.	Multi-model mechanistic
(L. Sun et al. 2025) [38]	Chondrocyte mitochondrial/innate-immunity model	Chondrocytes; experimental OA context	Piezo1 upregulation/activation; mtDNA and cGAS–STING pathway analysis	Elevated Piezo1 promoted mtDNA release and cGAS–STING activation, linking mechanotransduction to mitochondrial injury and innate immune signaling.	Mechanistic preclinical
(Yu et al. 2025) [13]	Mitophagy/mitochondrial-autophagy model	Chondrocytes; experimental knee OA model	Piezo1 pathway manipulation; mitochondrial autophagy assessment	Linked Piezo1 activation with mitochondrial autophagy dysfunction and cartilage injury, supporting impaired mitochondrial quality control in OA.	Mechanistic preclinical
(Y. Zhou et al. 2025) [39]	Metabolic–immune crosstalk model	Mesenchymal stem cells/Th17-cell OA model	Piezo1 mechanosensing; glycolytic pathway manipulation	Connected Piezo1-dependent metabolic reprogramming with MSC–Th17 crosstalk and OA progression.	Multi-model immunometabolic
(Y. Han et al. 2025) [51]	Chondrocyte signaling model	OA-relevant chondrocyte model	Piezo1 modulation; PI3K/AKT/mTORC1 pathway analysis	Supported a role for Piezo1 in chondrocyte homeostasis through PI3K/AKT/mTORC1 signaling and identified a potentially druggable downstream pathway.	In vitro/mechanistic preclinical
(Hu et al. 2023) [45]	Joint-tissue transcriptomic profiling	Rat OA model; multiple joint tissues	Longitudinal OA development; transcriptomic analysis	Identified dysregulated mechanotransduction and extracellular-matrix pathways across joint tissues during OA development.	In vivo + omics evidence
(Jurynec et al. 2026) [25]	Human genetics + single-channel functional analysis	Familial OA; primary human chondrocytes and synovial fibroblasts	PIEZO1 variant identification; electrophysiology; gene-expression analysis	Rare PIEZO1 variants associated with familial OA altered channel open probability; genetic and functional data indicated context-dependent effects of Piezo1 activity on OA susceptibility.	Human genetic + mechanistic
(Z. Yan et al. 2026) [8]	Synovial macrophage targeting + in vivo OA	Synovial macrophages; preclinical OA model	Macrophage-targeted Piezo1 siRNA delivery	Piezo1 knockdown reduced M1 polarization and suppressed the DRP1–cGAS–STING–NLRP3 axis, attenuating OA progression.	Multi-model preclinical
(Kan et al. 2026) [52]	Conditional knockout + human cartilage explants + OA models	Mouse; human cartilage explants	Excessive mechanical stress; Piezo1/Kdm5c genetic manipulation; telmisartan	Piezo1 activation promoted Kdm5c-dependent epigenetic silencing of cartilage-anabolic genes; genetic or pharmacological pathway inhibition protected cartilage.	Multi-model mechanistic + human ex vivo
(Y. Zhang et al. 2026) [46]	Biomaterial/regenerative OA model	Stem-cell-based preclinical OA model	Bioactive chitin-based thermosensitive hydrogel with stem-cell therapy	Local biomaterial support enhanced stem-cell-based cartilage repair, illustrating translational potential of intra-articular delivery platforms.	In vivo preclinical/biomaterial

Evidence categories are descriptive and do not correspond to a formal clinical hierarchy of evidence.

**Table 3:** Emerging Therapeutic Strategies Targeting the Piezo1–Mitochondria–Immune Axis in Osteoarthritis. Overview of the principal therapeutic approaches currently under investigation to modulate Piezo1 signaling, mitochondrial quality control, immunometabolic pathways, and structural joint degeneration in osteoarthritis.

Therapeutic Target	Representative Strategy	Primary Mechanism	Expected Biological Effects	Development Stage	References
Piezo1 inhibition	Genetic deletion, siRNA, small-molecule inhibition	Reduces pathological Ca^2+^ influx and mechanotransduction	↓ Chondrocyte apoptosis ↓ Matrix degradation ↓ Inflammatory signaling	Preclinical	[32,42,59]
GsMTx4	Mechanosensitive channel inhibitor	Attenuates mechanically induced Piezo1-dependent Ca^2+^ entry	↓ Ca^2+^ overload ↓ Ferroptosis ↑ COL2A1 and aggrecan expression	Experimental	[24,33]
Yoda1 (research tool)	Piezo1 agonist	Experimental activation of Piezo1 signaling	Mechanistic interrogation of Ca^2+^ signaling and mechanotransduction	Experimental model	[7,24,33]
Membrane lipid modulation	EPA/omega-3 polyunsaturated fatty acids	Modifies membrane properties and decreases Piezo mechanosensitivity	↓ Ca^2+^ overload ↓ Oxidative stress ↓ Chondrocyte apoptosis	Experimental	[3,4]
Mitochondrial protection	Antioxidant and mitochondrial-protective approaches	Limits mitochondrial oxidative stress downstream of mechanical/Piezo1 signaling	↓ ROS-associated injury Preservation of mitochondrial homeostasis	Preclinical/experimental	[8,28,33]
Mitophagy enhancement	PINK1/Parkin and PI3K–AKT–mTOR pathway modulation	Supports mitochondrial quality control and removal of damaged mitochondria	↑ Mitophagy ↑ Mitochondrial integrity ↓ Cartilage degeneration	Experimental	[13,25,41]
Mitochondrial dynamics modulation	DRP1 pathway modulation	Targets excessive mitochondrial fission and associated mitochondrial injury	↓ mtDNA release ↓ Innate immune activation	Experimental	[8,28]
Innate immune modulation	cGAS–STING pathway inhibition	Suppresses cytosolic mtDNA sensing and downstream innate immune signaling	↓ Synovial/chondrocyte inflammatory signaling ↓ Pro-inflammatory mediators	Experimental	[8,28]
Inflammasome inhibition	NLRP3 pathway inhibition	Limits inflammasome activation downstream of mitochondrial stress	↓ Inflammasome signaling ↓ IL-1β-associated inflammation Potential cartilage protection	Experimental	[8,28,33]
RNA-based therapies	Piezo1 siRNA/gene silencing	Selective suppression of Piezo1 expression	↓ Mechanotransduction ↑ Cartilage preservation	Preclinical	[8,49]
Biomaterial-based delivery	Injectable hydrogels	Enhances local retention and provides a protective biomechanical microenvironment	Improved local persistence Cartilage repair/regeneration support	Preclinical	[36]
Nanomedicine	Targeted lipid nanoparticles/nanocarriers	Cell-specific delivery of Piezo1-directed RNA therapeutics	Improved targeting Reduced systemic exposure Suppression of inflammatory signaling	Emerging/preclinical	[8]

Abbreviations: AKT, protein kinase B; Ca^2+^, calcium; cGAS, cyclic GMP–AMP synthase; COL2A1, collagen type II alpha 1 chain; DRP1, dynamin-related protein 1; EPA, eicosapentaenoic acid; GsMTx4, Grammostola spatulata mechanotoxin 4; IL-1β, interleukin-1 beta; mtDNA, mitochondrial DNA; mTOR, mechanistic target of rapamycin; NLRP3, NOD-like receptor family pyrin domain containing 3; PI3K, phosphoinositide 3-kinase; PINK1, PTEN-induced kinase 1; ROS, reactive oxygen species; siRNA, small interfering RNA; STING, stimulator of interferon genes; ↑, increased/activated; ↓, decreased/inhibited.

## Data Availability

No new data were created or analyzed in this study. Data sharing is not applicable to this article.
